# Developmentally-Dynamic Murine Brain Proteomes and Phosphoproteomes Revealed by Quantitative Proteomics

**DOI:** 10.3390/proteomes2020191

**Published:** 2014-04-03

**Authors:** Peter F. Doubleday, Bryan A. Ballif

**Affiliations:** Department of Biology, University of Vermont, 109 Carrigan Drive, Burlington, VT 05405, USA; E-Mail: pdoubled@uvm.edu

**Keywords:** brain development, phosphoproteomics, phosphorylation, quantitative mass spectrometry, reductive amination

## Abstract

Developmental processes are governed by a diverse suite of signaling pathways employing reversible phosphorylation. Recent advances in large-scale phosphoproteomic methodologies have made possible the identification and quantification of hundreds to thousands of phosphorylation sites from primary tissues. Towards a global characterization of proteomic changes across brain development, we present the results of a large-scale quantitative mass spectrometry study comparing embryonic, newborn and adult murine brain. Using anti-phosphotyrosine immuno-affinity chromatography and strong cation exchange (SCX) chromatography, coupled to immobilized metal affinity chromatography (IMAC), we identified and quantified over 1,750 phosphorylation sites and over 1,300 proteins between three developmental states. Bioinformatic analyses highlight functions associated with the identified proteins and phosphoproteins and their enrichment at distinct developmental stages. These results serve as a primary reference resource and reveal dynamic developmental profiles of proteins and phosphoproteins from the developing murine brain.

## 1. Introduction

Since its emergence two short decades ago, mass spectrometry-based proteomics has quickly matured to become not only a major force in discovery science, but also an important analytical tool in the testing of hypotheses formed in all manner of biochemical and biological disciplines [1]. Essential to the rapid maturation of proteomics have been remarkable advances in instrumentation, sample preparation methodology, and informatics. All of these have enabled proteomics to make major strides toward accomplishing two of its main goals: accurate protein identification and quantification at large or comprehensive scales [1]. Furthermore, significant progress has also been made on the identification and quantification of protein modifications. Dominant in these efforts has been the characterization of protein phosphorylation, owing to its recognized regulatory roles, the defined mass of its adduct, and what, in hindsight, can be considered relatively straight-forward approaches to enrich for phosphorylated peptide analytes [2].

Previously we conducted phosphoproteomic analyses of murine brain at embryonic day 16.5 (E16.5) using SCX [3]; neonatal (P0) brain using SCX-IMAC [4] and post-natal day 21 (P21) brain using anti-phosphotyrosine peptide immunoprecipitation [5]. These studies identified thousands of phosphorylation sites from primary tissue at distinct stages. Notably, when we compared our P0 SCX-IMAC dataset with a P21 dataset obtained in a near-identical fashion [6], we found that one third of the phosphorylation sites in the P0 dataset were not found in the P21 dataset even though the P21 dataset was more than twice the size [4]. These data are consistent with the intuitive hypothesis that phosphoproteomes are highly dynamic across development. Herein we document and quantitatively profile the changing proteomes and phosphoproteomes between three developmental stages of murine brain: E16.5, P0 and P21. These results will serve as a reference dataset facilitating the generation of many hypotheses relating to vertebrate brain development, ranging from orchestrated developmental control of protein cohorts to site-specific developmental regulatory mechanisms.

## 2. Experimental

### 2.1. Tissue Harvesting and Preparation of Tryptic Peptides

**Figure 1:**
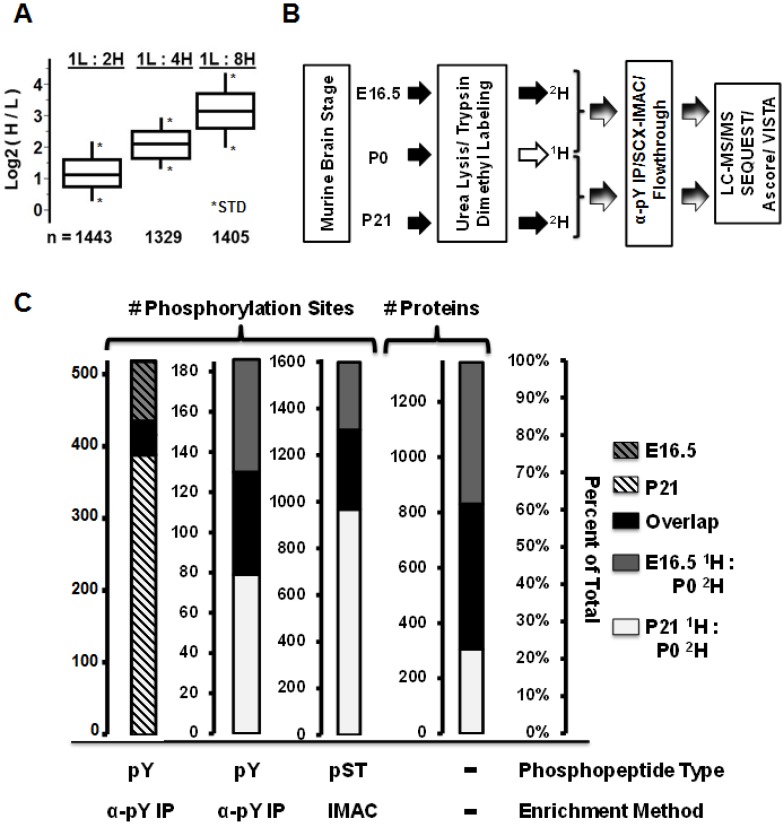
Workflow and overview of murine brain developmental proteomic and phosphoproteomic comparisons. (**A**) Reductive amination of tryptic peptides generates mass tags for quantitative proteomics. E16.5 tryptic peptides were subjected to reductive amination with either formaldehyde (CH_2_O) and sodium cyanoborohydride (NaBH_3_CN), or CD_2_O and NaBD_3_CN, to create light or heavy mass tags respectively. Light to heavy mixtures were made as indicated and subjected to LC-MS/MS, peptide identification and quantification. Approximately 1,400 peptides were identified and quantified. Box plots show mean, first and third quartiles and standard deviations; (**B**) Quantitative proteomics workflow used whole brains from E16.5, P0 or P21 mice that underwent urea lysis, trypsinization and differential dimethyl labeling as described above. 5 mg heavy-labeled tryptic peptides from E16.5 or P21 were each combined with 5 mg light-labeled P0. Mixtures were subjected first to anti-pY peptide IPs. Supernatants from the IPs were subjected to SCX-IMAC phosphopeptide enrichment. The flowthrough from the SCX-IMAC was also retained. The peptides bound to each affinity chromatography resin, as well as a portion of the unbound peptides were subjected to LC-MS/MS, SEQUEST-based peptide identification, Vista-based quantification and Ascore phosphorylation site evaluation as appropriate; (**C**) Summaries of phosphopeptide and protein identifications from our large-scale proteomic comparisons. The numbers of phosphotyrosine (pY) and serine/threonine phosphorylation sites (pST) and the number of proteins identified from non-phosphopeptides are provided as well as the percent of the total identified from each comparison. See text for details.

Mice were procured and treated in accordance with an institutionally-approved IACUC protocol. Timed-pregnant or appropriately-aged CD-1 mice were ordered from Charles River Canada (Saint Constant, QC, Canada). Timed-pregnant (E16.5), P0 and P21 mice were sacrificed and dissected after a brief isoflurane administration. Several brains from each stage were pooled and the pooled brains of a given stage were quickly weighed and placed on ice immediately prior to lysis. Three P21 brains were pooled, 8 P0 brains were pooled and 15 E16.5 brains were pooled. For dimethyl-labeling experiments, pooled brain tissue from each stage (~750 mg) was dounce-homogenized in a total of 25 mL ice-cold urea lysis buffer (8 M urea, 110 mM NaCl, 25 mM TRIS pH 8.0, 25 mM NaF, 10 mM Na_4_P_2_O_7_, 1 mM Na_3_VO_4_, 50 mM β-glycerolphosphate, 10 µg/mL leupeptin, 5 µg/mL pepstatin A and 1 mM PMSF). The homogenate was sonicated on ice for six 30-s intervals, with 30-s rests on ice between blasts, using a Kontes 50 W sonication microprobe tip at 50% duty output. Insoluble cellular debris was removed by centrifugation at 7,000× *g* for 30 min at 4 °C. DTT (5 mM final concentration) was added to 20 mL of the clarified lysate. The lysates were then incubated at 65 °C for 30 min. After cooling to room temperature, iodoacetamide was added to 12 mM and lysates were incubated for one hour at room temperature in the dark. Samples were diluted in 25 mM TRIS pH 8.0 to a urea concentration of 2 M. 750 µg of sequencing-grade of modified trypsin (Promega, Madison, WI, USA) was added to each 10 ml of lysate for overnight digestion at 37 °C. Peptides were acidified with trifluoroacetic acid (TFA) to 0.4% and insoluble material was spun down by centrifugation at 4,000× *g* for 20 min. The clarified supernatant from each developmental stage was applied to a separate, prewashed (100% acetonitrile (MeCN)) and equilibrated (0.1% TFA) tC18 (1 g) solid phase extraction Sep-Pak column (Waters, Milford, MA, USA). Peptides were washed with 0.1% TFA and eluted in 40% MeCN, 0.1%TFA. Eluates were frozen at −80 °C and lyophilized. For non-labeled peptides used in the anti-phosphotyrosine peptide immunoprecipitation experiment shown in Figure 1C, E16.5 murine brain was lysed in brain complex lysis buffer (BCLB; 25 mM Tris pH 7.2, 137 mM NaCl, 10% glycerol, 1% Igepal, 25 mM NaF, 10 mM Na_4_P_2_O_7_, 1 mM Na_3_VO_4_, 1 mM PMSF, 10 µg/mL leupeptin and 5 µg/mL pepstatin A). The crude homogenate was clarified by centrifugation at 15,000× *g* and at 4 °C. Eleven hand-poured 7.5%–20% acrylamide gradient, preparative gels containing 6 mg protein each were cut into four distinct molecular regions. Similar regions were combined and subjected to in-gel tryptic digestion prior to peptide extraction and peptide immunoprecipitations. A detailed description of peptide preparation in this manner was described previously [3].

### 2.2. Dimethyl Labeling of Tryptic Peptides

For the large-scale analysis, 10 mg (weighed) of dried tryptic peptides from each developmental stage were separately dissolved in 5.0 mL of 1 M HEPES, pH 7.5. 200 µL of fresh 4% D_2_-formaldehyde (Cambridge Isotopes Laboratories, Tewksbury, MA, USA) and 200 µL 600 mM NaCNBD_3_ (Cambridge Isotopes Laboratories) were added to both E16.5 and P21 peptides. 200 µL of 4% formaldehyde and 200 µL of 600 mM NaCNBH_3_ were added to P0 peptides. Reactions were allowed to proceed for 10 min, followed by a second 200 µL addition of each reagent and another 10 min incubation. Reactions were quenched by adding TFA to 10% and peptides were desalted over tC18 (0.5 g) solid phase extraction Sep-Pak columns as described above with the washing being 2.5% MeCN, 0.1% FA and the elution with 40% MeCN, 0.1% TFA. Peptide eluates were frozen and lyophilized. For the small-scale analysis shown in Figure 1A, ~2 µg of each mixture was subjected to LC-MS/MS analysis.

### 2.3. Anti-Phosphotyrosine Peptide Immunoprecipitations

^1^H-dimethyl P0 peptides, ^2^H-dimethyl E16.5 peptides, ^2^H-dimethyl P21 peptides were dissolved in 1.4 mL of peptide immunoprecipitation buffer (PIPB; 50 mM MOPS/NaOH pH 7.2, 10 mM Na_2_HPO_4_, 50 mM KCl). 0.7 mL (5 mg) of ^1^H-dimethyl P0 peptides was mixed separately with 0.7 mL (5 mg) of ^2^H-dimethyl P21 peptides or 0.7 mL or ^2^H-dimethyl E16.5 peptides. Peptides were rocked at 4 °C for 30 min and insoluble remnants were removed by centrifugation in a microcentrifuge (15,000× *g*) at 4 °C for 15 min. 30 µL of a 50% slurry of immobilized α-pY100 (Cell Signaling Technology, Danvers, MA, USA) was added to each mixed peptide solution. The solutions were rocked overnight at 4 °C and immune complexes were loaded onto a 200 µL gel-loading tip, pinched to arrest the resin. The resin was washed five times with PIPB and twice with water. The original flow through was collected for SCX-IMAC. Phosphotyrosine-harboring peptides were eluted with two applications of 40 µL of 0.15%TFA. The eluate was desalted on stage tips as previously described [5] and dried prior to LC-MS/MS. The non-quantitative E16.5 peptide IP was conducted as previously described [5] and used the monoclonal 4G10 α-phosphophotyrosine antibody (Upstate Biotech/Millipore, Billerica, MA, USA).

### 2.4. SCX-IMAC

The flow through from peptide IPs was subjected to SCX-IMAC as described previously [2,3] but the SCX was modified to use salt bumps rather than an HPLC as described [7]. Briefly, dried peptides were resuspended in SCX buffer A (7 mM KH_2_PO_4_ pH 2.65, 30% MeCN) and applied to a polysulphoethyl A solid phase extraction column (PolyLC Inc, Columbia, MD, USA) pre-washed with 80% MeCN and then with H_2_O, and then equilibrated with SCX buffer A for 30 min. Peptides were eluted fractionally with 6 mL each of SCX solvent B (7 mM KH_2_PO_4_ pH2.65, 30% MeCN, 350 mM KCl) adjusted with SCX solvent A to 0 mM, 10 mM, 25 mM, 40 mM, 60 mM, 90 mM and 150 mM KCl. Eluates were collected, frozen at −80 °C, lyophilized, and then desalted on tC18 (0.5 g) solid phase extraction Sep-Pak columns as described above and dried. 

### 2.5. Mass Spectrometry and Data Analysis

Dried peptides were suspended in 2.5% MeCN, 2.5% formic acid (FA) and loaded for nano-scale microcapillary LC-MS/MS in an LTQ-Orbitrap MS (Thermo Electron, Waltham, MA, USA) fitted to a Finnigan Nanospray II electrospray ionization source, a Surveyor HPLC pump plus, and a Micro AS autosampler (all from Thermo Electron) essentially as described [5]. Briefly, after an isocratic loading for 15 min in solvent A (2.5% MeCN, 0.15% FA) peptides were separated on an increasing MeCN gradient (2.5%–35%) with 0.15% FA from 15 to 60 min on a 100 µm internal diameter, in-house prepared 13 cm long MagicC18 reverse phase column (5 µm, 200 Å; Michrom Bioresources, Auburn, CA, USA) with a needle tip diameter of ~4.5 µm. Peptide measurements were identified in a top-10, data-dependent fashion, using the SEQUEST algorithm (Thermo Electron V26.12) against the mouse IPI database (mouse IPI v3.60) in a target-decoy approach [8], allowing for phosphorylation of serine, threonine, tyrosine (+79.96633 Da), oxidation of methionine (+15.99429 Da), carbamiodomethylation of cysteine (+57.02146 Da), and heavy mass addition to N-termini and lysine (+6.03766). Peptides were required to incorporate a static mass addition of N-termini and lysine residues through dimethyl labeling (+28.0313 Da). Quantification of heavy and light peptide pairs was accomplished by interrogating MS1 full scans and comparing integral values of the distinct isotopic envelopes for each peptide pair using Vista-based software [9,10]. MS runs from each developmental comparison were pooled by subset—non-phosphopeptides, serine and threonine phosphopeptides, and phosphotyrosine peptides—and initially filtered below a 1% false discovery rate using an automated linear discriminant analysis as previously described [6] weighted by Xcorr, ΔCn, MS2 ion intensity, missed tryptic cleavages, precursor PPM, and peptide length. Relative confidence in phosphorylation site localization was assessed using the Ascore algorithm [11]. Note that in some cases the Ascore program adjusted the site of phosphorylation from what was designated by SEQUEST.

### 2.6. Immunoblots and Antibodies

Whole brains from E16.5, P0 or P21 CD-1 mice (similarly pooled as described in Section 2.1) were dounce-homogenized in BCLB (see above). The crude homogenate was clarified by centrifugation at 15,000× *g* and at 4 °C. Normalized extracts were run on 7.5% or 10% (37.5:1 polyacrylamide/bis-acrylamide) SDS-PAGE gels to achieve separation. Immunoblotting was performed using 0.2 µm nitrocellulose membranes, blocked with 5% dry milk in Tris-buffered saline (TBST) essentially as described previously [12], and incubated in primary antibodies (α-CRMP2, α-GSK3β pY216 from Santa Cruz Biotechnology, Santa Cruz, CA, USA; α-CRMP2 pT514, α-DCX, α-DCX pS334 and α-β-Tubulin from Cell Signaling Technology) diluted in 1.5% bovine serum albumin (BSA) in TBST overnight at 4 °C. HRP-conjugated secondary antibodies (Chemicon/Millipore, Billerica, MA, USA) were diluted in TBST. Enhanced chemo-luminescence was performed prior to visualization by exposure to Hyblot CL film (Denville Scientific, Metuchen, NJ, USA).

### 2.7. Bioinformatics

DAVID [13,14] was utilized to generate enriched Gene Ontology categories, clusters and enriched KEGG [15,16] pathways from developmental comparisons utilizing IPI accessions as gene identifiers. Prior to conducting bioinformatics analyses, phosphopeptides or non-phosphopeptides from each developmental comparison were divided into separate sub-lists. Further, to investigate the contribution of quantitative changes to particular pathways and functional ontologies, phosphoproteins sub-lists were created from genes in the top and bottom 2.5% of each quantitative phosphopeptide dataset.

## 3. Results and Discussion

### 3.1. Comparative Murine Brain Proteomics Reveals Developmentally-Dynamic Proteomes and Phosphoproteomes

Previously we found major differences in the identified phosphoserine and phosphothreonine sites when comparing SCX-IMAC-enriched phosphopeptides from P0 and P21 murine brain [4]. To determine if major differences in tyrosine phosphorylation profiles could be similarly observed between developmental states, we conducted a large-scale anti-phosphotyrosine immuno-affinity enrichment from E16.5 murine brain and compared it to results we previously reported for P21 murine brain [5]. We found that of the 162 phosphotyrosine sites we identified from E16.5 brain only 64 (40%) were also found in our study of phosphotyrosine sites from P21 brain which identified 409 sites [5]. These results are graphed in Figure 1 and the phosphotyrosine sites identified from E16.5 murine brain are provided in Supplementary Table S1. Note we provide in Supplementary Document S1 expanded descriptions of technical terms used throughout Supplementary Tables.

While the identification of specific sites of phosphorylation at distinct developmental stages is important and helpful, the results would be stronger if measured in a more quantitative way. Therefore, we next aimed to monitor the developmental dynamics of the murine brain phosphoproteome, as well as the proteome generally, using a quantitative mass spectrometry workflow (Figure 1) involving reductive amination to introduce stable-isotope-based mass tags at primary amines in tryptic peptides [17]. Prior to conducting large scale comparisons, we first examined the general effectiveness of the approach on a smaller scale. E16.5 tryptic peptides were dimethylated in one case using formaldehyde and sodium cyanoborohydride without heavy labels, while in the other case the formaldehyde and sodium cyanoborohydride contained deuterium atoms. The end result of the heavy chemical reaction generated heavy-labeled peptides with six deuterium atoms per primary amine, thereby providing a mass tag distinguishable from light counterparts. We made 1:2, 1:4 and 1:8 (light:heavy) mixtures of the samples prior to subjecting them to LC-MS/MS, peptide identification and quantification. In each case roughly 1400 peptides were identified and quantified. Box plots of the Log2-transformed data including the means, the first and third quartiles, and the standard deviations are presented in Figure 1. These data show that the first and third quartiles for each mixture did not overlap, suggesting this method would provide in our hands relatively strong binary quantification capacity at least −/+ three Log2 orders.

We therefore proceeded with large-scale analyses using whole brain from three developmental stages, E16.5, P0 and P21. We used binary comparisons: E16.5 compared with P0 and P0 compared with P21. P0 peptides were light-labeled and used as the common reference, with E16.5 and P21 peptides heavy-labeled. As outlined in Figure 1, we first subjected the mixed peptide sets to anti-phosphotyrosine immunoprecipitations. The unbound peptides were subjected to SCX-IMAC. Both sets of affinity purified peptides, as well as the SCX-IMAC flow through, were desalted and subjected to LC-MS/MS and data analysis as detailed in the experimental section. 

Between the two binary comparisons, over 150 unique phosphotyrosine sites (Supplementary Tables S2 and S3) and over 1500 unique phosphoserine and phosphothreonine sites (Supplementary Tables S4 and S5) were identified and quantified. The identification overlap between the two binary comparisons was roughly 25% for each phosphorylation site dataset (Figure 1). This means that relative quantification at each of the three developmental stages is possible for 25% of the phosphorylation sites and relative quantification is possible at two developmental stages for 75% of the phosphorylation sites. These results are consistent with the distinct differences that we observed between developmental states in our non-quantitative comparisons discussed above. For protein identification and quantification, we used a minimum cutoff of three peptides. Roughly 40% of the over 1300 identified proteins were found in both binary comparisons enabling their relative quantification at each of the three stages (Figure 1). The quantified proteins and the individual non-phosphopeptides used for quantification are provided in Supplementary Tables S6–S8.

### 3.2. Immunoblotting of Developmentally-Important Phosphoproteins Shows Agreement with Quantitative MS Data

**Figure 2:**
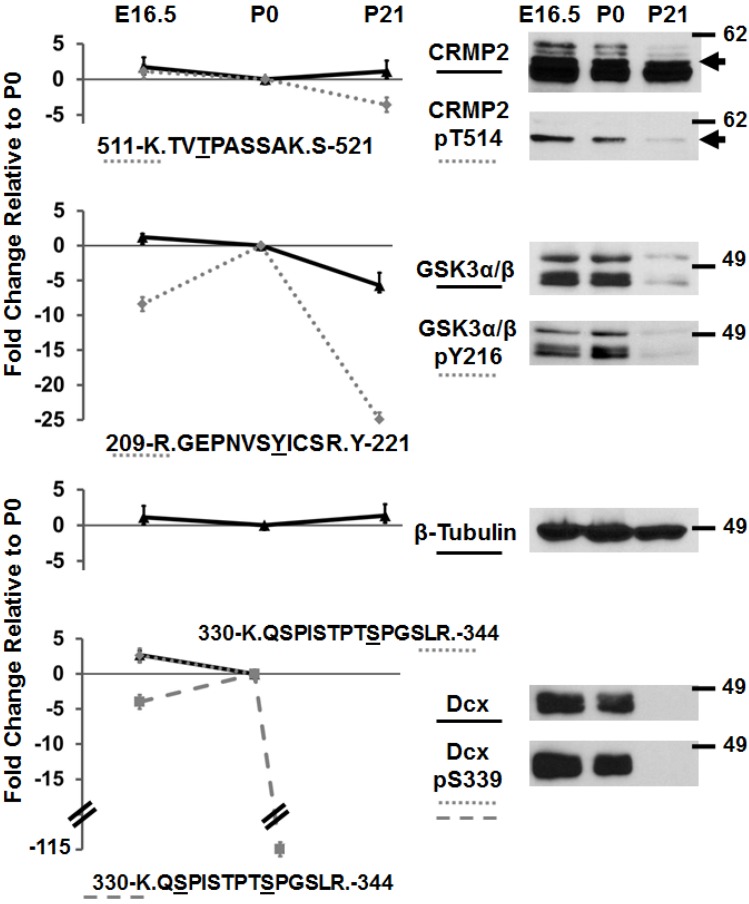
Immunoblots parallel quantitative mass spectrometry results. Immunoblots and quantitative mass spectrometry were conducted as described in the experimental section, and represent the protein and phosphospecific protein levels of the indicated proteins at the indicated developmental state. Graphical data on the left represent the fold changes of proteins and indicated phosphopeptides between each developmental state as determined by mass spectrometry. The sites of phosphorylation are underlined. Errors bars for the mass spectrometry data represent the standard deviations of the mean for all non-phosphorylated tryptic peptides identified from the indicated protein. Error bars for the phosphopeptides represent the standard deviations of the mean from all individual measurements of the indicated phosphopeptide. Arrowheads at the top right represent the same molecular weights. Molecular weight standards in daltons are indicated.

Although while still relatively few in number, several specific phosphorylation events have been shown to be critical for proper vertebrate brain development as elaborated on previously [2,3,4,5,12,18,19]. As an orthogonal approach by which to evaluate the quantitative mass spectrometry data, we used immunoblotting to sample a few proteins and a few specific phosphorylation sites known to be critical in brain development. We examined Collapsin Response Mediator Protein (CRMP2) and its phosphorylation at T514 (Figure 2). CRMP2 phosphorylation by Glycogen Synthase Kinase-3 (GSK-3) at T514 follows an initial priming phosphorylation by Cyclin-dependent Kinase 5 (CDK5) and these phosphorylation events play important roles in neuronal migration in response to guidance cues [20]. We also examined by immunoblotting the levels of GSK-3 and its activation loop phosphorylation at tyrosine 216, the levels of tubulin, and the critical regulator of brain development Doublecortin (DCX) and DCX phosphorylation at S339, presumably by CDK5 [21]. All of the immunoblotting results are presented in Figure 2 and are accompanied by quantitative graphs generated by the mass spectrometry data of these same proteins and phosphorylation sites. The agreement between the immunoblots and the quantitative mass spectrometry is strong. The DCX results also parallel results we obtained and reported previously using absolute quantification (AQUA) mass spectrometry [22] as well as immublotting [4].

### 3.3. Proteomes and Phosphoproteomes Display Distinct Profiles at Different Stages of Brain Development

**Figure 3:**
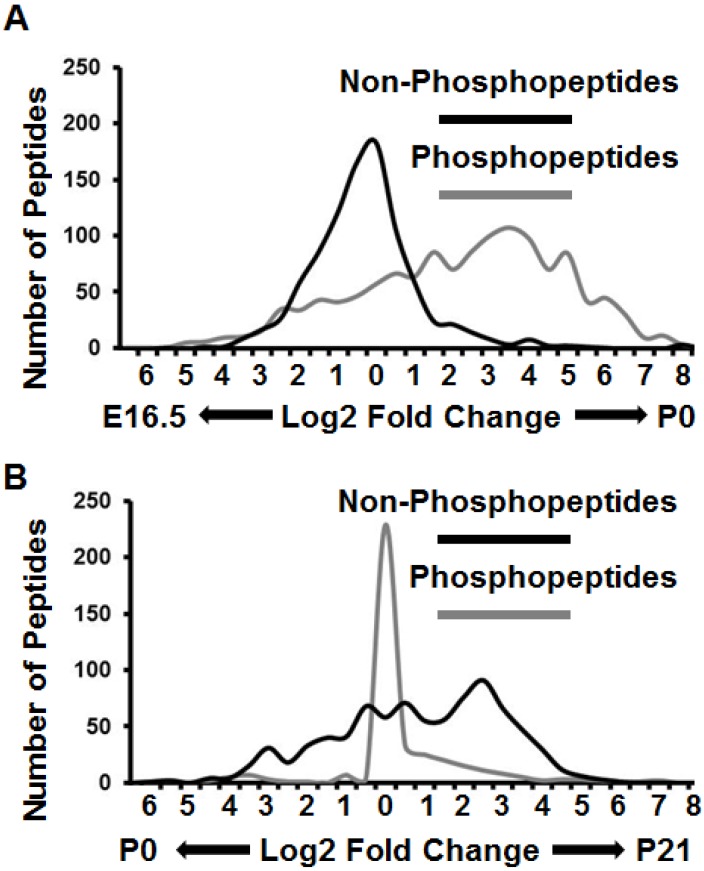
Quantitative mass spectrometry results show dynamic proteomic and phosphoproteomic profiles at distinct developmental stages of the murine brain. (**A**) Plot of the E16.5:P0 datasets; (**B**) Plot of the P0:P21 datasets. Phosphopeptides and non-phosphopeptides were placed into 0.5 Log2 fold-width bins and the numbers in each bin were plotted as a smoothened line.

Graphing Log2 fold changes of the phosphopeptides and the non-phosphopeptides between each developmental comparison, we were surprised to find distinctly different profiles. We found that for the non-phosphopeptides the fold change between E16.5 and P0 brain formed a curve with tighter Gaussian characteristics as compared to the phosphopeptides, whose fold changes were more spread and leaned toward higher abundances at P0 (Figure 3). In contrast, the phosphopeptides in the comparison between P0 and P21 showed tight Gaussian characteristics with the non-phosphopeptides being more spread and leaning slightly toward higher abundances at P21 (Figure 3). These results clearly show the highly dynamic proteomes and phosphoproteomes across brain development and surely represent the sum of the multitude of regulatory mechanisms occurring at these stages including differential transcription, translation, and cell differentiation. The more uniform phosphopeptides between P0 and P21 may reflect that tissues types are differentiating less between these stages and phosphorylation events are therefore more drivers of homeostasis than developmental processes. 

### 3.4. Phosphotyrosine Sites Showing the Most Extreme Differences between E16.5 and P21 Brain Offer Avenues for Distinct Hypothesis Testing

Large-scale phosphoproteomic analyses have come of age and now greatly out-pace our ability to examine the functional consequences of individual phosphorylation events, not to mention cohorts! However, curation of phosphorylation sites, as is done at a multitude of sites such as PhosphositePlus [23] and Prosite [24] offers investigators interested in conducting functional studies, opportunities to envisage hypotheses of mechanistic operation. Table 1 shows the phosphotyrosine sites showing the largest (11 fold or more) difference in the relative abundance between E16.5 and P0 as well as between P0 and P21. Each one of these sites has been identified in dozens, and in some cases hundreds, of large-scale studies. However, in PhosphositePlus only two are reported to be functionally characterized: Dab1 pY232 which we found previously to be essential for Dab1’s interaction with Crk/CrkL in Reelin signaling [18], and GSK3 pY216 which has been extensive characterized as the activating event in GSK3’s activation loop. The large, observed changes in these phosphotyrosine sites invites investigation into their potential roles governing vertebrate brain development.

**Table 1:** Phosphotyrosine Sites with Largest Fold Change Quantified between E16.5 and P0 and P0 and P21 Murine Brain.

**Gene Symbol**	**pY Site**	**Peptide (Y# is pY)**	**Log2 Fold Higher E16.5 over P0**	**Fold Higher E16.5 over P0**	**Protein Name**
Mbp	199	TTHY#GSLPQK	6.61	98	Myelin basic protein
Dlg4	283	NTYDVVY#LK	6.35	81	Discs large homolog 4, Postsynaptic density protein 95
Syt1	229	TLVMAVY#DFDR	5.51	45	Synaptotagmin I
Grin2b	1039	HSQLSDLY#GK	5.39	42	GluRepsilon2/ N-methyl D-aspartate receptor 2B
Dlg2	340	HMLGEDDY#TRPPEPVYSTVNK	4.92	30	Discs large homolog 2, Postsynaptic density protein 93
Syt1	380	VFVGY#NSTGAELR	4.54	23	Synaptotagmin I
Ckmt1	154	SGY#FDER	4.53	23	Creatine kinase, mitochondrial 1
Mbp	169	GAY#DAQGTLSK	3.64	12	Myelin basic protein
Dlg4	647	FIEAGQY#NSHLYGTSVQSVR	3.50	11	Discs large homolog 4, Postsynaptic density protein 95
**Gene Symbol**	**pY Site**	**Peptide (Y# is pY)**	**Log2 Fold Higher P0 over P21**	**Fold Higher P0 over P21**	**Protein Name**
Dab1	232	EGVY#DVPK	3.91	15	Disabled 1
Prpf4b	338	LCDFGSASHVADNDITPY#LVSR	3.99	16	Pre-mRNA-processing factor 4b
Afap1l2	413	VAQQPLSLVGCDVLPDPSPDHLY#SFR	4.21	18	Actin filament-associated protein 1-like 2
Hipk3	359	TVCSTY#LQSR	4.58	24	Homeodomain interacting protein kinase 3
Gsk3b	216	GEPNVSY#ICSR	4.64	25	Glycogen synthase kinase-3 beta
Hipk1	352	AVCSTY#LQSR	5.23	38	Homeodomain interacting protein kinase 1

### 3.5. Bioinformatics Reveals Functional and Developmental Dynamics in Brain Proteomes and Phosphoproteomes

**Figure 4:**
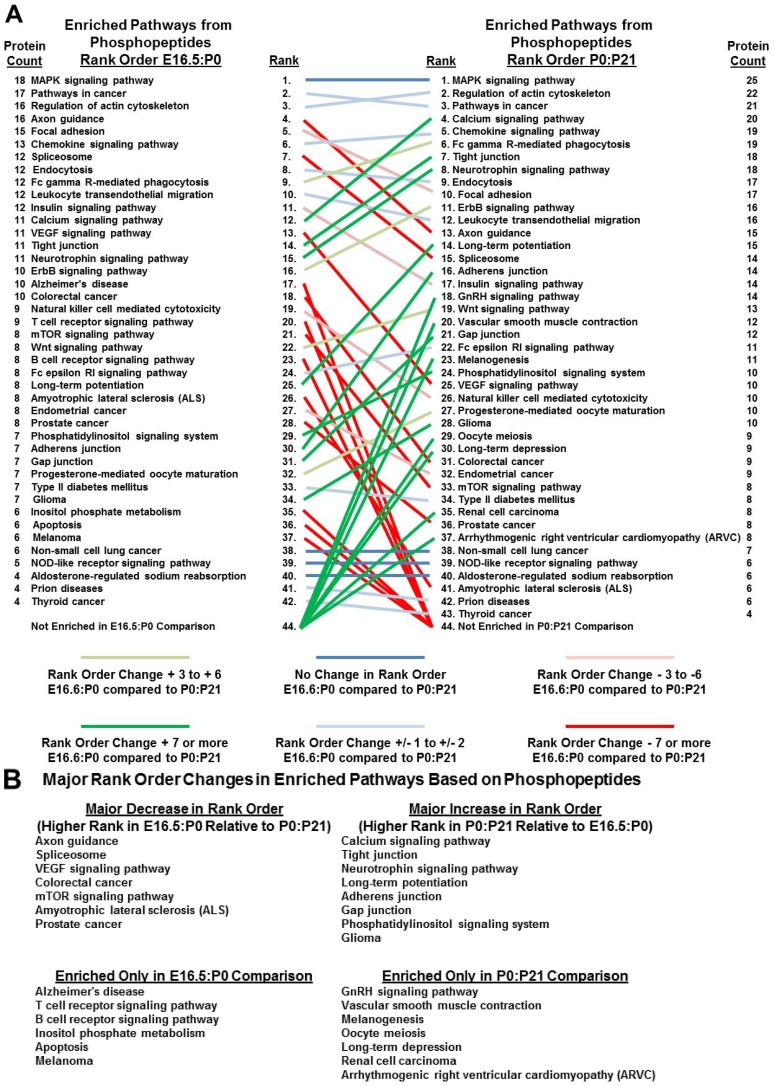
(**A**) Rank order differences in enriched KEGG pathways from proteins identified in E16.5:P0 and P0:P21 phosphopeptide datasets indicate potential functional developmental dynamics; (**B**) Major differences (>6 or <6) in rank are summarized.

**Figure 5:**
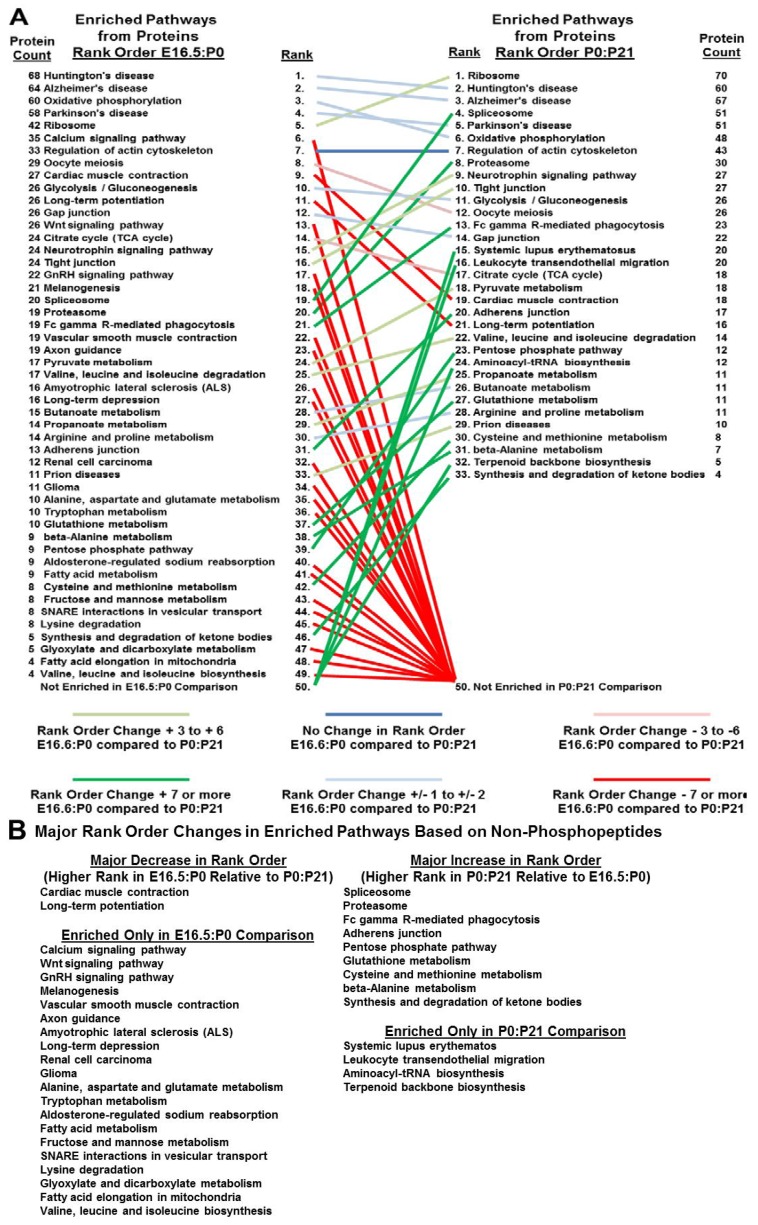
(**A**) Rank order differences in enriched KEGG pathways from proteins identified in E16.5:P0 and P0:P21 non-phosphopeptide datasets indicate potential, functional developmental dynamics; (**B**) Major differences (>6 or <6) in rank are summarized.

To determine if specific functional pathways based on protein counts were overrepresented relative to the mouse proteome in either our brain phosphopeptide dataset or our non-phosphopeptide dataset we queried the Database for Annotation, Visualization and Integrated Discovery (DAVID) to obtain Kyoto Encyclopedia of Genes and Genomes (KEGG) [15,16] pathway data. The analysis identified several pathways that were enriched in the E16.5:P0 dataset and the P0:P21 datasets. The enriched pathways, unique identifiers for the proteins in these pathways, and relevant KEGG statistics are presented in Supplementary Tables S9 and S10. The enriched pathways showed important differences comparing developmental stage and when considering non-phosphopeptide and phosphopeptide datasets, with some pathways enriched exclusively in only one dataset. A mechanism to visualize such presence/absence binary differences as well as differences where an enriched pathway is found in both datasets but its relative enrichment competes in magnitude with other enriched pathways is a simple rank order analysis. To accomplish this we ranked the enriched pathways based on the number of protein counts and visualized differences in rank order by drawing lines between the enriched pathway from the E16.5:P0 dataset to the same enriched pathway in the P0:P21 dataset. The slopes of the lines, as well as their color enhancement, accentuate rank order differences (Figure 4 and Figure 5).

Enrichments offer insight into active pathways, particularly when high protein counts (or high rank) are considered. We recognize that a simple rank order discounts various weighting options including weighting by protein counts or by fold enrichment. However, these approaches can also be conducted using the data in Supplementary Tables S9 and S10. Still, this simple rank order approach is useful in showing variable forms of enrichment in our datasets. First, some pathways are only enriched at the phosphopeptide level. For example, Mitogen-activated protein kinase (MAPK) signaling and cancer signaling pathways were the most dominantly-enriched pathways in both developmental phosphopeptide datasets. However, MAPK signaling and cancer signaling pathways were not enriched in the non-phosphopeptide datasets. This suggests these proliferation-based pathways exhibit significant phospho-regulation at all three of these stages of brain development, even if their protein levels do not show enrichment compared to brain phosphoproteomes generally. Second, several pathways show enrichment at both the phosphopeptide and non-phosphopeptide levels. This includes one of the next most highly-ranked pathways, regulators of the cytoskeleton. This pathway also exhibits strong enrichment and relatively-high ranking in the non-phosphopeptide datasets. However, axon guidance pathways are most strongly enriched in the E16.5:P0 phosphopeptide dataset and are only enriched in the P0:P21 non-phosphopeptide dataset. This is not surprising given the requirement for axon guidance mechanics during neural development. Finally, those enriched pathways showing strong changes in rank order, or enrichment in only one dataset, offer insight into bias in developmental dynamics. One example, in addition to the axon guidance pathway already discussed, is the spliceosome. Splicing interestingly is only enriched in the E16.5:P0 phosphopeptide dataset, but conversely only enriched in the P0:P21 non-phosphopeptide dataset. This suggests strong developmental phospho-regulation of the spliceosome, but an increase in total spliceosome protein representation post-development, perhaps for routine splicing. Developmentally-regulated splicing has been appreciated for some time [25,26].

The KEGG analysis described above focused on enrichments found in entire datasets. However, given we had acquired quantitative mass spectrometry information, and given our primary focus on developmentally-regulated phosphorylation events, we next wanted to determine the nature of the phosphoproteins that we had identified and which had a relative abundance importantly higher in one developmental stage over another. To do this we took our Log2 fold change-sorted datasets described in Figure 3 and reduced them to phosphoproteins that were quantitatively in the top or bottom 2.5% of the data. These proteins were used to query DAVID to look for clusters of gene ontology categories not necessarily exclusively pathways (Supplementary Table S11, which includes the relevant protein identifications and DAVID statistics). These data for both phosphopeptide datasets are shown in Table 2 which abbreviates the data to summarize the major differences observed.

Consistent with developmentally-dynamic phosphoproteomes these DAVID clustering results identify differential enrichment in gene ontology categories when analyzing the phosphopeptides that exhibit the largest changes in abundance between brain stages. Importantly, both the KEGG pathways and other DAVID clustering results are tied to distinct proteins and in some cases distinct phosphorylation sites which will facilitate the generation of hypotheses that lead to the characterization of proteins with putative roles in vertebrate brain development.

**Table 2:** DAVID gene ontology summary of phosphoproteins showing major fold changes in the quantitative mass spectrometry-based phosphopeptide comparisons.

**Enriched Gene Ontology Categories of Phosphoproteins Showing Major Fold Changes in the E16.5:P0 Phosphopeptide Comparison**	
**Enriched in E16.5**	**Enriched in P0**
Cytoskeleton	Protein Complexes Cytoskeleton Non-Membrane Bound Organelles
Neuronal Projection	
Synaptic Transmission	
Cell Signaling	
Splicing	
**Enriched Gene Ontology Categories of Phosphoproteins Showing Major Fold Changes in the P0:P21 Phosphopeptide Comparison**	
**Enriched in ** **P0**	**Enriched** **in P21**
Neurogenesis and Differentiation	Synaptic Transmission
Developmental Growth	Ion Transport
Morphogenesis	Cognition
Cell Signaling	Cell Signaling
Various Metabolism	Non-Membrane Bound Organelles

## 4. Conclusions

We have conducted a large-scale, quantitative mass spectrometry study of murine brain at three developmental stages: E16.5, P0 and P21. The relative abundances for thousands of phosphorylation sites and proteins across these developmental stages were determined. Bioinformatic analyses identified phosphoproteins and proteins from each developmental stage with enrichments tied to distinct functionality. Several of these functionalities are consistent with nervous system development and development generally. However, much remains unexplored and it is anticipated that these results will serve as an important resource for developmental biologists as well as for cellular biochemists, particularly those studying the roles of phosphorylation on individual proteins or protein cohorts.

## Supplementary Materials

Supplementary File 1
