# Supplementary material for: Developmentally-Dynamic Murine Brain Proteomes and Phosphoproteomes Revealed by Quantitative Proteomics
Source: Proteomes. 2014 Apr 3;2(2):191–207. doi: 10.3390/proteomes2020191 (PMC4146493; doi:10.3390/proteomes2020191)
Supplement: Supplementary File 1 [file proteomes-02-00191-s001.zip › proteomes-48417-supplementary-final/proteomes-48417-supplementary-final.pdf]

Supplementary Material

Document 1

<u>Expanded Descriptions of Terms Found in Supplementary Tables:</u>

z: Charge state of the analyte in mass spectrometry analyses. Peptides need to be ions to be mobilized and analyzed within the electromagnetic fields of mass spectrometers. Masses are calculated by mass spectrometers as mass/charge (*m/z*). Tryptic peptides typically hold two protons (or a charge state of +2) after ionization procedures in acidic solvents given the presence of amine functions at the N- and C-termini. However, lower or higher charge states can also occur depending on peptide length, amino acid functional groups *etc*.

PPM: Or (parts per million) is a measurement facilitating an assessment of how close the observed (or measured) mass matches the theoretical (or expected) mass of the analyte. It is calculated as:

$$\text{PPM} = [(\text{m theoretical} - \text{m observed})/\text{m theoretical}] \times 10^6$$

Most high resolution mass spectrometers yield PPM values for peptide analytes that are +/− 5 PPM. Note that as mass increases PPM values can increase.

XCorr: XCorr or cross-correlation scores are scores generated by the SEQUEST algorithm when computing how well a given tandem mass spectrum correlates to a peptide sequence found in the protein database being searched. Higher scores denote stronger matches or correlations. Higher scores are achieved largely due to the observed presence of fragment ions in the tandem mass spectrum which was generated when peptides were subjected to chemical and/or physical dissociations. Of note, the higher the charge state (*z*), the more possible fragment ions, and thus there is a higher likelihood of achieving a higher XCorr value. Thus longer peptides will therefore have the potential for higher XCorr values. Note that greater confidence in how meaningful an XCorr value is can be achieved by searching concatenated databases of all proteins in forward and reverse orientation. These concatenated database searches using PPM, XCorr and other parameters such as ΔCn values (discussed below) help define false-discover rates, given that the reverse orientation tryptic sequences are almost all (0.01%) not overlapping with correct orientation tryptic sequences at ten amino acids [1]. In large-scale studies, it is rare that a XCorr score above 3.5 will be found among false-positive reverse sequence hits [1].

ΔCn: This is a SEQUEST measure that is used to determine how close the top two XCorr values are, and therefore can give some measure of confidence in the top sequence match generated by SEQUEST. It is calculated as 1 − (XCorr of the second best match/XCorr of the best match). Small values suggest there is little confidence that the top match is a better match than the second best match. Larger values (typically above a value in the 0.15–0.20 range) provide added confidence in the correct assignment to the top hit.

Unique ΔCn: In some cases, particularly when considering post-translational modifications, the second best hit may have an identical amino acid sequence to the first hit but with only the site of modification altered. For example, a SEQUEST best match might be the peptide GYFDDLAS#SVR (with S# denoting phosphorylation). This peptide will likely have the second best match GYFDDLASS#VR and perhaps the third best match as GY#FDDLASSVR. The second and third best matches are the same peptide sequence with different phosphorylation site assignments. In this case the XCorr between the

first match and the second match is likely very low, and might be removed in a filter that required a ΔCn greater than or equal to 0.15. So, a "unique ΔCn" or a "ΔCn2" can be used which will perform the ΔCn calculation but only use the next best match of a different peptide sequence.

IPI: International Protein Index [2]. Note that the IPI website and database remains static at the moment as funds have not been invested for its maintenance (see website for details).

Redun: This is the number of other entries in the database that have the same exact peptide sequence or redundancy. Thus, a "2" in the "Redun" column means that two other entries (likely splice variants or homologs) have an exact sequence match to the one found to be the SEQUEST top match.

Ascore: Whenever phosphorylation is identified on a peptide with multiple phosphorylatable residues (STY in this consideration), it is possible that the SEQUEST assignment is not accurate or perhaps ambiguous. Various programs have been generated to attempt to determine if the phosphorylation site is correctly assigned. We have used the Ascore program [3] to generate an ambiguity score for any phosphorylation site assignment. Scores above ~13 are generally considered to be correctly assigned. One can have an Ascore for as many phosphates found to be on the peptide.

Site: This is the amino acid number in the linear (N- to C-terminal) sequence of amino acids in the particular database entry. In this case it refers to the phosphorylation site.

Log2 Heavy/Light Area: To calculate the relative abundance between a heavy and light peptide pair one can use the maximum peak height of the two peptides as they elute off the column and are measured in the mass spectrometer. An alternative approach uses the area under the curves of the eluting peptides. The latter is generally considered to be preferred whenever the heavy label contains deuterium as deuterium can sometimes lead to slight shifts in retention time on the reverse phase chromatography column. However, reductive amination of primary amines, as used here, typically does not lead to important shifts in peptide retention time [4]. Log2 transformation is helpful in compressing data in visual space and it also transforms data to reduce distortion in various downstream analyses particularly when datasets contain both numbers above one and numbers with decimals between zero and one. For example, if a heavy peptide is four times the relative abundance of the light peptide the untransformed (heavy/light) calculation will be 4. If the light peptide is four times the heavy then the untransformed (heavy/light) will be 0.25. Log2 transformed data would yield +2 and −2 respectively.

Sum S:N: Signal to noise (S:N) is the signal above the background. In isotope-based quantitative mass spectrometry the sum of the S:N of the light and heavy peptide analytes speaks, at least to one degree, to the confidence one can have in the quantification. If the detection (and subsequent quantification) is not well above the noise then less confidence can be put in the quantification. The sum of the S:N above 10 makes quantification significantly stronger [5].

Spectral Count: The number of times a particular peptide was identified by tandem mass spectrometry.

References

1. Elias, J.E.; Gygi, S.P. Target-decoy search strategy for increased confidence in large-scale protein identifications by mass spectrometry. *Nat. Methods* **2007**, *4*, 207–214.
2. International Protein Index. Available online: http://www.ebi.ac.uk/IPI (accessed on 14 March 2014).
3. Beausoleil, S.A.; Villen, J.; Gerber, S.A.; Rush, J.; Gygi, S.P., A probability-based approach for high-throughput protein phosphorylation analysis and site localization. *Nat. Biotechnol.* **2006**, *24*, 1285–1292.
4. Khidekel, N.; Ficarro, S.B.; Clark, P.M.; Bryan, M.C.; Swaney, D.L.; Rexach, J.E.; Sun, Y.E.; Coon, J.J.; Peters, E.C.; Hsieh-Wilson, L.C., Probing the dynamics of o-glcnac glycosylation in the brain using quantitative proteomics. *Nat. Chem. Biol.* **2007**, *3*, 339–348.
5. Bakalarski, C.E.; Elias, J.E.; Villen, J.; Haas, W.; Gerber, S.A.; Everley, P.A.; Gygi, S.P., The impact of peptide abundance and dynamic range on stable-isotope-based quantitative proteomic analyses. *J. Proteome Res.* **2008**, *7*, 4756–4765.

© 2014 by the authors; licensee MDPI, Basel, Switzerland. This article is an open access article distributed under the terms and conditions of the Creative Commons Attribution license (http://creativecommons.org/licenses/by/3.0/).
